# Phenotypic Plasticity Provides a Bioinspiration Framework for Minimal Field Swarm Robotics

**DOI:** 10.3389/frobt.2020.00023

**Published:** 2020-03-16

**Authors:** Edmund R. Hunt

**Affiliations:** ^1^Department of Engineering Mathematics, University of Bristol, Bristol, United Kingdom; ^2^Bristol Robotics Laboratory, University of the West of England, Bristol, United Kingdom

**Keywords:** phenotypic plasticity, reaction norms, swarm diversity, resilience, minimal robotics, swarm robotics

## Abstract

The real world is highly variable and unpredictable, and so fine-tuned robot controllers that successfully result in group-level “emergence” of swarm capabilities indoors may quickly become inadequate outside. One response to unpredictability could be greater robot complexity and cost, but this seems counter to the “swarm philosophy” of deploying (very) large numbers of simple agents. Instead, here I argue that bioinspiration in swarm robotics has considerable untapped potential in relation to the phenomenon of phenotypic plasticity: when a genotype can produce a range of distinctive changes in organismal behavior, physiology and morphology in response to different environments. This commonly arises following a natural history of variable conditions; implying the need for more diverse and hazardous simulated environments in offline, pre-deployment optimization of swarms. This will generate—indicate the need for—plasticity. Biological plasticity is sometimes irreversible; yet this characteristic remains relevant in the context of minimal swarms, where robots may become mass-producible. Plasticity can be introduced through the greater use of adaptive threshold-based behaviors; more fundamentally, it can link to emerging technologies such as smart materials, which can adapt form and function to environmental conditions. Moreover, in social animals, individual heterogeneity is increasingly recognized as functional for the group. Phenotypic plasticity can provide meaningful diversity “for free” based on early, local sensory experience, contributing toward better collective decision-making and resistance against adversarial agents, for example. Nature has already solved the challenge of resilient self-organisation in the physical realm through phenotypic plasticity: swarm engineers can follow this lead.

## Introduction

The self-organized societies of social insects such as ants are well-known in swarm robotics (Şahin, 2005); yet they could be the “tip of the iceberg” of available bioinspiration. Here, I focus specifically on the general concept of *phenotypic plasticity* as a powerful, complementary framework for thinking about real-world deployment of minimal robot swarms. In fact, social insects are prime exhibitors of phenotypic plasticity (Kennedy et al., 2017), but it is widespread and of fundamental importance in the rest of the natural world. In brief, I argue the following main points:

1. Plasticity is typically selected for by evolution following a natural history of unstable environmental conditions. In offline evolutionary swarm optimization, simulated environments need to be more heterogeneous and hazardous to generate and understand the value of plasticity.
2. In the context of large numbers of agents, elements of this plasticity could be (partially) irreversible, as in nature. This could be further enabled by cost-effective expendability, up to and including recyclable or biodegradable robots.
3. In addition to the value of individual plasticity for responding to environmental variation, otherwise unremarkable variation in response thresholds (for example) can contribute to adaptive group-level diversity; swarm engineers can exploit this.

I first provide some background perspective on swarm robotics before introducing the biological phenomenon of phenotypic plasticity.

### Background: The “Swarm Principle” of Individual-Level Simplicity

Swarm robotics is predicated on the idea that large numbers of agents working collectively can solve tasks that would be impossible for a single individual (Hamann, 2018). It is specifically inspired by biology in that it relies on self-organization (Camazine et al., 2001) as the mechanism of coordination, particularly as seen in social insects (Şahin, 2005). This includes concepts such as stigmergy (e.g., Hunt et al., 2019a). Closely allied to this is the reliance on *emergence* of swarm problem-solving capabilities that cannot be reduced to, or predicted from, individual-level components (Şahin, 2005; Bjerknes et al., 2007; Brambilla et al., 2013).

As technology continues to develop, with ever-advancing computer processing power and methods in artificial intelligence, the temptation may be to build swarms of agents that are individually highly complex both in their hardware and controllers. However, this would not align with the “swarm principle” of relying on emergence to do the “heavy lifting” of solving the task. It would also defeat the object in “complexity engineering” of maintaining low-level understandability (Frei and Giovanna, 2012). Finally, it may be prohibitive in terms of cost, when real-world environments have hazards resulting in a risk—or even an expectation—of robots being lost or destroyed. Instead, swarm controllers are classically based on reactive control (Hamann, 2018), based on simple reflexes to a stimulus (e.g., Walter, 1950; Mitrano et al., 2019), or taking into account an internal state (the model-based reflex agent of Russell and Norvig, 1995, for example Nouyan et al., 2009). This “behavior-based robotics” (Arkin, 1998) is in keeping with studies of reaction thresholds in biology (Bonabeau et al., 1999). It is also compatible with relatively simple and affordable hardware that can be easily understood: for example the “e-puck” (Mondada et al., 2009), “Kilobot” (Rubenstein et al., 2012), and “Crazyflie” (McGuire et al., 2019). There is still relatively limited real-world swarm deployment (e.g., Schmickl et al., 2011; Duarte et al., 2016): there is a clear opportunity to shape the design principles for minimal swarms.

### Previous Examples of Adaptation in Homogeneous Robot Swarms

There are several examples in the swarm robotics literature in which individual robots, though identically programmed with the same controller, end up behaving differently according to their experience of the environment. I briefly group these according to three prominent approaches, before going on to explain the complementarity of the proposed approach.

#### Off-Line (Pre-deployment) Evolutionary Optimization

**Figure 1:**
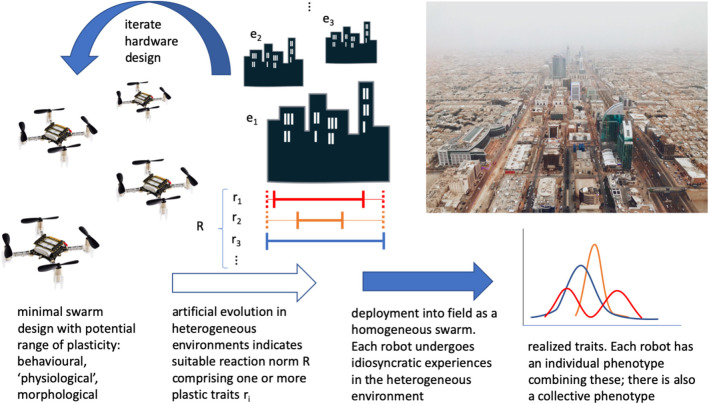
A conceptual overview of how phenotypic plasticity could be employed in a minimal robot swarm. Beginning with existing minimal robot hardware, consider the current and potential extent of plasticity. Undertake artificial evolution of swarms in a series of heterogeneous environments, to obtain suitable developmental reaction norms (mappings of sensory input to ranges/variations of phenotype, including one or more variable traits such as reaction thresholds, power consumption, or “smart” body parts). Hardware may be iterated to extend or reduce/remove plasticity. Deploy into the field, and individual robot experience will contribute to a distribution of individual phenotypes in the swarm. This should then form an adaptive swarm-level phenotype. Robots can then be collected and reset before redeployment elsewhere, recycled/disposed of sustainably, or even biodegrade in certain contexts (“Crazyflie” drone photo CC-BY 4.0, Bitcraze AB).

Designing emergent (Matarić, 1993) and adaptive (Matarić, 1995) group behaviors is challenging, and so one can use evolutionary optimization in simulation before deployment (Dorigo et al., 2004; Trianni, 2008; Hecker and Moses, 2015; Birattari et al., 2019). In this way, adaptation of behavior can be seen in task specialization, for example, as an effective group-level strategy (Ferrante et al., 2015), though its effectiveness is tuned to the particular simulated environment. Furthermore, the simulated environments employed in evolutionary robotics can be rather simple and homogeneous. As a result, there can be little in the way of a mechanism to generate plasticity, as it is not rewarded by the artificial evolutionary process. Including sufficient heterogeneity in the class of simulated environments is indispensable to identifying a suitable variety and extent of plasticity for swarm robots (Figure 1).

#### On-Line (On-Deployment) Evolutionary Optimization

Embodied evolutionary robotics is a promising avenue for real-world deployment (Trueba et al., 2011; Haasdijk et al., 2014; Jones et al., 2019) but in practice the requisite computing power may be a step away from the minimal robotics needed for swarm ubiquity. Evolutionary approaches (off- or on-line) could struggle in the field, owing to unanticipated circumstances or merely because of the so-called “reality gap” between the world and (inner) simulation (Brooks, 1992; Jakobi et al., 1995).

#### Learning (On-Deployment)

Learning is an example of behavioral plasticity. For example, if one simulates improved task performance through repetition there can be emergent task specialization (Brutschy et al., 2012). Task sequencing has been demonstrated at run-time without prior knowledge of the correct ordering, demonstrating a form of reinforcement learning, albeit with abstractions of the tasks themselves (Garattoni and Birattari, 2018). In practice, robot learning tends to employ (evolved) neural networks (Nolfi et al., 1994; Floreano and Mondada, 1996; Nolfi and Floreano, 2000; Nitschke et al., 2012; Hüttenrauch et al., 2018), so-called neuro-evolution methods. Neural network-based approaches can have difficulty in scaling to more complex problems (Brambilla et al., 2013); and again, for truly minimal swarms, this may be a step toward undue computational complexity. I suggest “personality” adaptation as an example minimal bioinspired approach to learning (section Behavioral Plasticity).

### Phenotypic Plasticity: Evolving Adaptive Reaction Norms

**Figure 2:**
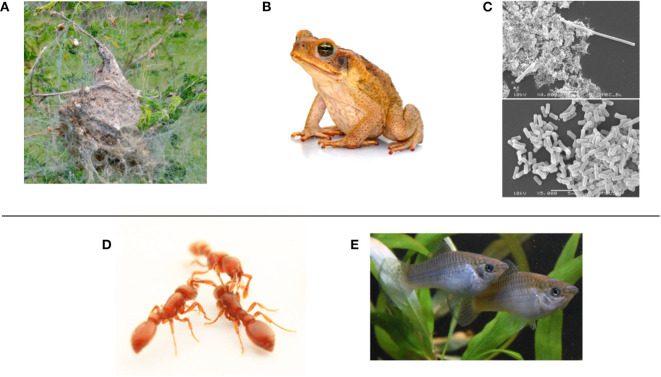
Top row: Examples of phenotypic plasticity, which could be enacted with simple, environmentally-reactive control rules. They are ordered left to right in the typical speed of response. **(A)** Behavioral plasticity in the social spider *Stegodyphus dumicola* allows the colony to maintain a suitable distribution of “bold” and “shy” individuals (photo: Bernard Dupont, CC-BY-SA 2.0). **(B)** Physiological plasticity is observed in the invasive cane toad *Rhinella marina*, as it can adjust its core temperature to live in cool regions (photo: Sam Fraser-Smith, CC-BY-2.0). **(C)** Morphological plasticity in the bacterium *Bacillus cereus*. In the top pane it has undergone filamentation following antibacterial treatment; bottom pane is untreated (photo: Achara Dholvitayakhun, CC BY-SA 4.0). Bottom row: Biologists study clonal organisms to understand how phenotypic plasticity produces individual differences within social groups (“swarms”). The distribution of differences may be adaptive for their local ecology. The individual-level “interaction rules” and resultant plasticity used in their self-organization may be instructive for swarm robot controllers. **(D)** The clonal raider ant *Ooceraea biroi* (photo: courtesy of Daniel Kronauer). **(E)** the Amazon molly *Poecilia Formosa* (photo: courtesy of David Bierbach).

Broadly defined, phenotypic plasticity is the ability of an organism's genotype to produce different phenotypes in response to different environmental conditions (Kelly et al., 2012). This includes behavioral, physiological, and morphological plasticity as I later describe in their respective sections (see also Figure 2). These are ordered by how rapidly an adjustment is typically made through that plasticity mode. Plasticity varies, as we see in social insects: some are resilient to environmental change (e.g., invasive ants; Holway et al., 2002), while others such as bees struggle to cope with e.g., habitat loss, novel toxins, or pathogens (Goulson et al., 2015). Its importance may in part depend on mobility: for instance, it is particularly important in plants, which are unable to change their environment (Schlichting, 1986). Early experience is often key to phenotypic development (e.g., Weaver et al., 2004), which can be seen as a form of “memory” of the environment to which the organism (or agent) is exposed in the initial phase of its life (deployment).

The term developmental reaction norm (DRN) describes the range of phenotypes generated by a given genotype (“controller,” smart materials, etc.) in response to experienced environmental cues (Schlichting and Pigliucci, 1998). DRNs can themselves be plastic or non-plastic, i.e., the phenotype can remain fixed or change in response to changing environmental conditions. Therefore, there are at least five attributes to DRNs: amount of plasticity (large/small); pattern of response (e.g., monotonic increase/decrease or more complex reaction curves); rapidity of response; reversibility of response; and competence (possibility) of the developmental system to respond at a certain stage in an organism's (robot's) lifetime (Schlichting and Pigliucci, 1998). Moreover, in the “swarm” context, it is worth noting that individuals' experiences can affect the extent of their plasticity at a given age (Stamps, 2016). This can also contribute to group-level diversity in phenotypic expression. Behavioral plasticity at the level of the whole group can be seen in, for example, the reaction thresholds of harvester ant colonies (Gordon et al., 2011). In social groups individual phenotypes interact, contributing to the complexity of the genotype and phenotype fitness landscapes (Moore et al., 1997; Wolf et al., 1999). The various attributes of developmental reaction norms are, in principle, subject to natural selection (Schlichting and Pigliucci, 1998; Dingemanse et al., 2010), and I propose that for swarm engineers, pre-deployment artificial evolution of DRNs can establish their extent (Figure 1). Plasticity occurs in response to environmental cues, so one must also consider the relevant environmental features (physical and social) that will elicit change—and how they will be sensed. For example, local cues about resource distributions can be used to adjust individuals' foraging parameters (Just and Moses, 2018), and environmental heterogeneity generates variable foraging rates through behavioral plasticity in harvester ants (Beverly et al., 2009).

### Emerging Technologies Favoring (Partially) Irreversible Plasticity

In the context of model-based reflex behaviors, if internal reaction thresholds are computer variables there is no design requirement to make their setting irreversible; though this may be suitable for time and geography-limited missions, where robots can be retrieved and reset for redeployment. Several emerging technologies favor irreversible plasticity, however. For example, the field of “soft” robotics employs soft structures to flexibly interact with unpredictable environments (Kim et al., 2013). Robot intelligence can be “outsourced” from the computer “brain” to the robot “body” (morphology) and its nonlinear responses, exploiting “embodied intelligence” (Bongard, 2011). This outsourcing can go a step further in collectives, as phenotypic diversity in soft swarms could result merely from past sensitivity (hysteresis) to exposure temperature, strain and other conditions. Moreover, soft robots raise the possibility of biodegradability (Rossiter et al., 2016), further relaxing constraints on ubiquitous deployment. Another exciting development is the possibility of “autonomous” or “robotic” materials (McEvoy and Correll, 2015), smart composites that can autonomously change shape, stiffness, appearance and other properties. In electronics, the idea of a “memristor”—a resistor with “memory” of the charge flowing through it—raises the possibility of “neuromorphic computing” that parallels in some way the synaptic plasticity of a brain (Zidan et al., 2018; Wang et al., 2019). At smaller length scales, exciting possibilities exist for micro-scale swarms (e.g., Martel et al., 2009; Yigit et al., 2019). As robot swarms aim toward large numbers, and possibly smaller scales, the heterogeneity and stochasticity associated with minimal robots may become inevitable. Rather than seeing this as an engineering nuisance, swarm designers can embrace its possibilities (White et al., 2004; Ramachandran et al., 2018; Scholz et al., 2018; Li et al., 2019), and (partially) irreversible plasticity could contribute toward adaptation to field conditions.

## Swarm-Level Strength in Individual-Level Diversity

Phenotypic plasticity can produce helpful individual-level adaptations: for example, a suitable threshold to switch behaviors. Even more significantly in a swarm context, though, is the possibility of producing emergent functionality for the group. Even in what appear to be superficially similar units in cooperative biological groups there can be a surprising level of diversity (Blodgett et al., 2016); this heterogeneity is increasingly recognized as an adaptive group trait (Clobert et al., 2009; Kennedy et al., 2017). Thus, while plasticity in a certain trait may actually make a small or negligible contribution to the direct fitness of the individual, it may be nevertheless an important indirect contribution to the fitness of the swarm.

### Diversity as a Shield Against Adversity

Robustness is frequently claimed for swarm robot systems, but if a homogeneous controller results in homogeneous behavior the swarm may be liable to systematic failure if it encounters unexpected environmental conditions or faulty or malicious agents (Higgins et al., 2009). This might be compared to inbreeding in biology, which is a cause of disease vulnerability. Conversely, diversity can help resistance (Ugelvig et al., 2010).

Fault tolerance in swarms is an important precondition for scalability (Winfield and Nembrini, 2006; Bjerknes and Winfield, 2013) and phenotypic plasticity may paradoxically help the swarm to cope with the unexpected. This is because it can result in a range of subtle—or substantial—individual differences, which will need to be made compatible with agent—agent interaction as a matter of course.

### Diversity for Homeostasis

In biological systems phenotypic diversity can also promote positive collective success: for example in honeybees diversity in reaction thresholds for their cooling behavior promotes stability in nest thermoregulation (Jones et al., 2004). Although this example is driven by genetic heterogeneity, it could equally be designed in a robot context as a result of phenotypic plasticity.

### Diversity for Decision-Making

If a swarm is to be autonomous it also needs to be capable of making collective decisions. Again, diversity of reaction thresholds or option assessment behavior, as seen in ants, may help this process (Masuda et al., 2015; O'Shea-Wheller et al., 2017). Such studies highlight the importance of heterogeneity among individuals, rather than precise calibration, for effective collective decision-making.

### Diversity for Foraging and Search

Finally, variation in individual behavior can be important for foraging and search in systems as diverse as ants and immune systems (Beverly et al., 2009; Fricke et al., 2016).

## Behavioral Plasticity

Behavioral plasticity allows organisms to make relatively rapid adjustments in their function to adapt to changing environmental conditions. Learning, which shapes behavior, can be seen as a form of plasticity (Agrawal, 2001) and allows “culture”—inter-generational transmission of behaviors through social learning (Whiten et al., 2017). In robot swarms this has been demonstrated in robot societies through imitation learning (Winfield and Erbas, 2011), and can arise simply from robot and sensor noise (Erbas et al., 2013). Perhaps the most obvious opportunity for ready transposition into robot swarms, though, is seen in animal “personality” differences.

### Animal and Robot “Personalities”

Modeling work in biological collective behavior often assumes agents are homogeneous in their characteristics, but there is increasing recognition that consistent individual differences in behavior (“personality”) among group members can be important for group function in local ecologies (Dall et al., 2012). Examples of significant personality axes include: risk-taking behavior (boldness—shyness), exploratory behavior (neophilic—neophobic), activity levels (active—inactive), sociability (social—asocial), and aggression (aggressive—non-aggressive) (Réale et al., 2007). This can be observed at the level of the individual or the whole group, giving rise to the notion of collective personalities (Jandt et al., 2014). While early development is important to the formation of personality, it can be somewhat plastic over an individual's lifetime (Groothuis and Trillmich, 2011). As a result, group-level plasticity in personality is also observed (Norman et al., 2017). In *Stegodyphus* social spiders (Figure 2A), there is a link between social interactions and boldness change (Hunt et al., 2018); the group-level distribution of boldness is important for their collective performance (Hunt et al., 2019b).

In relation to swarm robotics, the notion of personality maps readily to adaptive threshold-based behaviors, for example the likelihood of switching behaviors in probabilistic finite state machines (Liu and Winfield, 2010; Castello et al., 2016). It can also map to very simple adaptations such as variable waiting times in response to changing swarm density (Wahby et al., 2019), which one might term “sociability,” for example. Simpler still, the decision to be active or inactive, which may make little sense at the level of the individual robot with a mission to complete, can be adaptive to a swarm that might need to keep some units in reserve; the identification of “lazy ants” (Charbonneau and Dornhaus, 2015) suggests plasticity in activity may be valuable. Thus, the growing literature on animal personality research—particularly on its ontogeny in social groups—may indicate simple behavioral mechanisms (“interaction rules”) that can be adapted in the context of self-organizing robots.

### The Relevance of Highly Related and Clonal Animals

In social insects, caste determination (e.g., worker or queen) is driven by a varying combination of “nature” (genotype) and “nurture” (environment) (Schwander et al., 2010). To try and understand how the environment (particularly the social environment) shapes such phenotypic plasticity, biologists study highly related or even clonal organisms, which controls for the effect of genetics. Social spiders (Figure 2A) are highly inbred; and two emerging model organisms are the clonal raider ant *Ooceraea biroi* (e.g., Ulrich et al., 2018) and the Amazon molly *Poecilia Formosa*, a small freshwater fish (e.g., Bierbach et al., 2017) (Figures 2D,E). As well as being prime candidates to answer fundamental questions in ecology and evolution (Laskowski et al., 2019), such organisms could provide important bio-inspiration to the development of homogeneous swarm controllers that can result in heterogeneity that is adaptive at the swarm-level.

## Physiological and Morphological Plasticity

An example of physiological plasticity in nature is the invasive cane toad *Rhinella marina* (Figure 2B). It succeeds as an invader into unfamiliar environments, at least in part, because it can adjust its core body temperature to new climates (McCann et al., 2018). It is also somewhat plastic in its social behavior (Gruber et al., 2017): an example of successfully combining multiple modes of plasticity. Physiological plasticity in a robotics context could mean something as simple as the availability of different power consumption modes: for example, a high energy mode for exploration and data transmission, and a standby mode for *in situ* monitoring of an environment. This could be critical to long-term swarm resilience.

Examples of morphological plasticity in nature include the water flea *Dapnia lumholtzi* (Green, 1967), which can respond drastically to the presence of predators by developing a sharp helmet and extended tail spine (Agrawal, 2001); or in bacteria that undergo filamentation (elongation) in response to stress (Figure 2C; Justice et al., 2008). At the group level, a form of collective mechanical adaptation is observed in honeybee swarms (Peleg et al., 2018). In swarm robotics research so far, a form of morphological plasticity is possible through self-assembly into connected groups of various forms (Brambilla et al., 2013). Examples of this include the “s-bot” which can physically attach to each other (Mondada et al., 2004), conceptual demonstrations in “Kilobots” (Rubenstein et al., 2014; Slavkov et al., 2018; Carrillo-Zapata et al., 2019), or the idea of a “mergeable nervous system” (Mathews et al., 2017). More broadly, one can design robots to adapt their own morphology (Divband Soorati et al., 2019; Hauser, 2019; Kriegman et al., 2019); in combination such “multi-robot organisms” (Levi and Kernbach, 2010) may self-organize a wide range of adaptations.

## Discussion

Swarm robotics relies on the power of emergence to produce engineered systems that are capable of “more than the sum of their parts”. This is possible even with very simple agents. As we take robot swarms into the field, the temptation may be to move away from the principle of individual-level simplicity in hardware and controllers. Instead, a different way forward may be to re-focus on the ingenuity of nature in building resilient social systems. Increasingly, phenotypic plasticity is recognized as center-stage in producing adaptive biological variation, and would seem to be similarly indispensable in embodied collective artificial intelligences. We can, and should, attempt intensive off-line optimization of swarm controllers (Birattari et al., 2019), but this could be combined with possibilities to manifest plasticity in behavior, “physiology” and morphology in heterogeneous simulated environments. Their respective impact on swarm-level functions might be analyzed with respect to information flow (Pitonakova et al., 2016). In a “bottom-up” approach to swarm design (Crespi et al., 2008) a moderate amount of plasticity across these modes could be added with very limited cost, but potentially far-reaching implications for swarm resilience, contributing toward the practical realization of “dependable swarms” (Winfield et al., 2004).

For biologists, robots can be used as tools for understanding biological evolution (Doncieux et al., 2015). The systematic addition of various forms of “phenotypic plasticity” to robots could also contribute toward this aim. Meanwhile, for engineers, with plasticity and mass-producible minimal robots, the approach of sending large numbers of cheap and expendable units on missions (“fast, cheap and out of control”; Brooks and Flynn, 1989) might have a better chance of success. A review across plasticity modes and relevant organisms (e.g., for air, water or land) could become a routine part of a swarm design process. The symbiosis between biology and engineering seen in the field of swarm robotics can go from strength to strength.

## Data Availability Statement

The original contributions presented in the study are included in the article, further enquiries can be directed to the corresponding author.

## Author Contributions

EH conceived of this perspective and wrote the paper.

### Conflict of Interest

The author declares that the research was conducted in the absence of any commercial or financial relationships that could be construed as a potential conflict of interest.
